# Cross-Group EEG Emotion Recognition Based on Phase Space Reconstruction Topology

**DOI:** 10.3390/e27101084

**Published:** 2025-10-20

**Authors:** Xuanpeng Zhu, Mu Zhu, Dong Li, Yu Song

**Affiliations:** Tianjin Key Laboratory for Control Theory and Applications in Complicated Systems, School of Electrical Engineering and Automation, Tianjin University of Technology, Tianjin 300384, China; zxp20001025@126.com (X.Z.); zm78792021@163.com (M.Z.)

**Keywords:** electroencephalogram (EEG), emotion recognition, phase space reconstruction, cross-group

## Abstract

Due to the interference of artifacts and the nonlinearity of electroencephalogram (EEG) signals, the extraction of representational features has become a challenge in EEG emotion recognition. In this work, we reduce the dimensionality of phase space trajectories by introducing local linear embedding (LLE), which projects the trajectories onto a 2-D plane while preserving their local topological structure, and innovatively construct 16 topological features from different perspectives to quantitatively describe the nonlinear dynamic patterns induced by emotions on a multi-scale level. By using independent feature evaluation, we select core features with significant discrimination and combine the activation patterns of brain topography with model gain ranking to optimize the electrode channels. Validation of the SEED and HIED datasets resulted in subject-dependent average accuracies of 90.33% for normal-hearing subjects (3-Class) and 77.17% for hearing-impaired subjects (4-Class), and we also used differential entropy (DE) features to explore the potential of integrating topological features. By quantifying topological features, the 6-Class task achieved an average accuracy of 77.5% in distinguishing emotions across different subject groups.

## 1. Introduction

Emotion recognition has gradually become one of the core research directions in artificial intelligence and psychological science. As an important characteristic of human psychological activities, emotional states profoundly affect individual cognitive mechanisms, behavioral patterns, and decision-making choices. Accurate emotion recognition plays a key role in several application scenarios, including intelligent interaction systems, clinical diagnosis and assessment, personalized teaching, and intelligent driving [1].

Two models are currently available to characterize the complexity of emotions: the dimensional model and the discrete model. The dimensional model characterizes emotional states by establishing a multivariate coordinate system, like the V–A (valence and arousal) model [2], the V–A–D (valence, arousal, and domain) model [3], and the emotion wheel model [4]. The discrete model categorizes emotions into a number of base categories, and typical division patterns contain three categories (negative, neutral, and positive), four categories (sad, neutral, happy, and afraid), and six categories (sad, happy, surprised, afraid, angry, and disgusted) [5].

Emotion recognition can be categorized into explicit and implicit methods. Explicit methods usually rely on individual self-reports or behavioral observations, through questionnaires, facial expression analysis, or voice emotion analysis [6]. Although these methods are direct, they may be interfered with by individual subjective factors or situational influences [7]. In contrast, implicit methods such as EEG, electrocardiogram (ECG), and electrical skin response provide a more objective and reliable way of assessing emotion by directly measuring physiological signals. EEG signals reflect the synchronized electrical activity of post-synaptic potentials in our brain’s neurons, which not only captures changes in emotion, but also reflects the brain’s complex responses during emotional processing [8].

Recent studies on EEG emotion recognition have focused on two main areas: feature extraction and feature classification. Jin et al. [9] proposed a method based on empirical mode decomposition (EMD), which suppresses noise by decomposing signals into intrinsic mode functions and lays the foundation for emotion feature extraction. Wang et al. [10] used differential entropy matrices as features and adopted a 2D-CNN-LSTM classification model, achieving over 91% accuracy in both valence and arousal. Sha et al. [11] developed a graph-adaptive semi-supervised learning method that identifies the significant contributions of temporal, prefrontal, and parietal channels of emotion recognition, thereby enhancing classification performance through channel subset optimization. Qiu et al. [12] proposed a semi-supervised, fine-tuning graph attention network (SFT-SGAT), which employs a two-stage training strategy and improved the generalization performance of the EEG emotion recognition model.

However, traditional feature extraction methods, which rely on time domain, frequency domain, time–frequency domain, and spatial domain features, often depend on linear analysis techniques. These methods fail to fully capture the nonlinear characteristics and dynamics of EEG signals [13]. Phase space reconstruction, as one of the nonlinear methods, transforms the original time series into phase space by two parameters, time delay and embedding dimension, thus providing richer feature information and revealing the complex nonlinear patterns and dynamic changes of EEG signals [14]. Yan et al. [15] extracted multi-band persistent homologous features in the phase space of EEG signals for an emotion classification task. Yao et al. [16] applied phase space reconstruction to capture the nonlinear dynamics of EEG signals, and further combined it with complex network analysis to extract features for classifying negative, neutral, and happy emotions. Most of the above work focuses on the quantification of a single or small number of dynamic attributes, and lacks the systematic integration of multidimensional dynamic features in the phase space, which fails to comprehensively characterize nonlinear patterns and leads to the loss of key dynamic information.

At present, the most commonly used EEG emotion datasets include DEAP [17], MANNOB-HCI [18], and SEED [19], and the participants are all normal-hearing people. Unlike normal-hearing people, who are able to express their emotions through verbal communication, hearing-impaired people primarily interpret the emotional state of their communication partners through facial expressions and body language [20]. Communication barriers or misunderstandings can increase the risk of anxiety and depression among those with hearing impairments due to long-term difficulties in emotional recognition and expression. Hence, we built an EEG emotion dataset of hearing-impaired subjects by studying their performance under different emotional stimuli [21].

In this study, we propose an EEG emotion recognition scheme based on phase space reconstruction to construct emotionally discriminative features by mining the dynamic nonlinear properties of signals, and conducted subject-dependent experiments on the SEED and our hearing-impaired EEG dataset (HIED). The main contributions of this paper are as follows:

(1) After optimizing time delay and the embedding dimension, the high-dimensional phase trajectory is constructed. Local linear embedding (LLE) is introduced to reduce the trajectory dimension to 2-D. By maintaining the local relative positional relationship, the original high-dimensional structural information is effectively retained.
(2) We extract 16 topological features based on consideration of three perspectives (area of the trajectory, degree of distortion of trajectory edge, and distance of the trajectory point from the coordinate center), which reflects the dynamic changes and complex structure of the EEG signals.
(3) Quantitative analysis is conducted on the emotion-related topological features of normal-hearing people and hearing-impaired people. A cross-group 6-Class task is designed to explore the universality of the emotion recognition model and provide a direction for constructing a more compatible emotion analysis framework.

The rest of the paper is structured as follows: Section 2 lists the related works on EEG signals feature extraction, Section 3 describes the preparation of the experimental dataset, Section 4 describes signal preprocessing as well as the process of constructing the topological features for phase space reconstruction, Section 5 provides the results of the experiments, and Section 6 provides a discussion. Finally, the work is summarized in Section 7.

## 2. Related Work on Feature Extraction

### 2.1. Linear Features

In previous studies, the methods for extracting linear features of EEG signals have mainly focused on the time, frequency, and spatial domains. For time domain feature extraction, Jenke et al. [22] used mean, variance, standard deviation, and peak as EEG features for emotion recognition. Liu et al. [23] extracted the statistical parameters of EEG signals as features in the time domain, including the mean and standard deviation of the original signal. For frequency domain feature extraction, Vecchiato et al. [24] used power spectral density (PSD) to analyze frontal EEG activity changes while participants viewed commercial videos, with a focus on EEG asymmetry associated with pleasurable experiences. Zhou et al. [25] combined brain maps constructed by PSD and spatial interpolation algorithms to extract EEG representative features. Ozerdem et al. [26] assessed both positive and negative emotions by channel selection preprocessing and extracting features using discrete wavelet transform. For spatial domain feature extraction, Naser et al. [27] used dual-tree complex wavelet packet transform for feature extraction on EEG signals, combined with functional connectivity and graph theory measures, to classify different emotions. Yao et al. [28] constructed a functional brain network using clustering coefficients to classify positive, negative, and neutral emotions. Wu et al. [29] extracted three features from EEG-based functional connectivity networks: strength, clustering coefficients, and eigenvector centrality, which were input into the Deep Canonical Correlation Analysis (DCCA) model during classification. The above work shows that the linear feature extraction method has been widely applied, but it may not be able to fully capture the nonlinear dynamic characteristics of EEG signals.

### 2.2. Nonlinear Features

Due to the complex nonlinear dynamics of EEG signals, researchers have gradually turned their attention to the application of nonlinear analysis methods. Tuncer et al. [30] proposed a fractal pattern-based feature extraction method and utilized SVM for a four-classification emotion recognition task. Yang et al. [31] utilized nonlinear features such as the Lyapunov exponent and fractal dimension to validate model assessment for a four-classification experiment under subject-independent conditions. Han et al. [32] extracted differential entropy (DE) features and input them into a deep learning model combining multiscale convolution and TimesNet to obtain better classification results. In existing studies, some nonlinear features mostly focus on quantifying the local dynamic properties of EEG signals, and lack a systematic characterization of the global evolution rule of the dynamic system. In this paper, we transform EEG signals into multidimensional trajectories using phase space reconstruction. We then develop topological features to fully capture the nonlinear patterns of emotion from a combined geometric and dynamic perspective.

## 3. Preparation of the Experiment

The SEED Dataset was created by the BCMI lab, Shanghai Jiao Tong University. It has been widely used in studies of EEG emotion recognition and contains three kinds of emotion: sad, neutral, and happy [16]. A total of 15 Subjects (7 males and 8 females, with an average age of 23.27 years old) were recruited and asked to watch emotional videos (sad, neutral, and happy) and their EEG signals were collected simultaneously. We used 15 video segments (5 segments for each emotion), each of which was approximately 4 min long, with 45 s for self-assessment and 15 s for rest between videos. The experiment was conducted using a 62-channel ESI Neuroscan system for data collection.

In this paper, we constructed an HIED based on the SEED [33]. The HIED includes 12 males and 3 females, with an average age of 22.25 years old. In order to avoid the effects of different cultural backgrounds, the equipment, paradigm, and parts of movie clips (sad, neutral, and happy) selected for the experiment were the same as the SEED. The fear movie clips were instead selected by graduate students in psychology, who rated and selected the top 5 clips from a pool of candidate fear-inducing videos. The duration of all clips ranged from 3 to 4 min. Subjects signed an informed consent form approved by the Ethics Committee of Tianjin University of Technology before the start of the experiment (Approval No. TJUT-2021-EC-007). Table 1 shows the details of the selected movie clips.

## 4. Data Preprocessing and Feature Extraction

**Figure 1:**
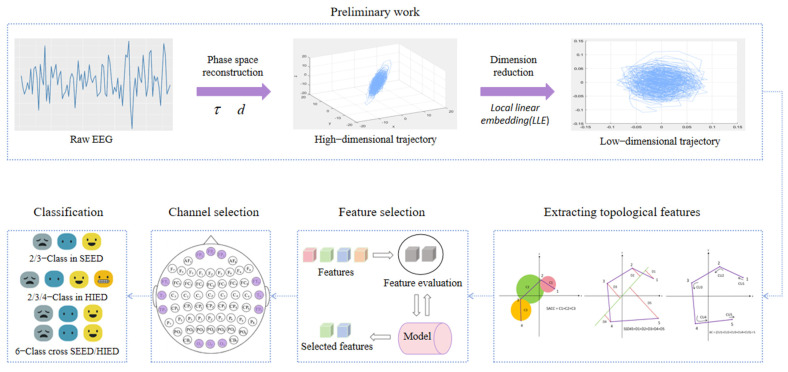
The main framework of the model. EEG signals were first reconstructed in phase space, then reduced to 2D by LLE, and 16 topological features were constructed from trajectory. Key features and channels were selected through analysis. At last, intra-group and cross-group emotion classification was validated on SEED and HIED datasets.

The main framework of the model is shown in Figure 1. The preprocessed EEG signals were reconstructed in phase space to generate high-dimensional trajectories, which were downscaled to the 2D plane by local linear embedding (LLE), preserving the local structure, and 16 topological features were extracted based on the emotionally correlated geometrical differences in the trajectories. Subsequently, key features and EEG channels were selected to optimize model efficiency and performance through independent feature analysis, brain topography activation patterns, and electrode channel gain assessment. Finally, the validation of intra-class emotion classification and six cross-group classification tasks were completed on the SEED and HIED, respectively.

### 4.1. EEG Signals Preprocessing

The raw EEG signals were downsampled to 200 Hz to ensure the efficiency of data processing. We applied a 1–75 Hz bandpass filter to isolate the main frequency band associated with emotions and a 49–51 Hz notch filter to eliminate powerline interference, followed by the interpolation of invalid channels, and finally performed independent component analysis (ICA) to reject eye blink artifacts. In order to ensure the validity and consistency of the analysis, as well as to avoid fatigue, distraction, or other external interfering factors affecting the results, we selected the last 180s of data for further feature extraction and analysis. In order to fully represent the differences in mapping trajectories in phase space and to ensure a sufficient number of samples, a non-overlapping time window of 9 s was selected as each segment [34].

### 4.2. The Construction of the DE Feature

In this paper, in order to explore the potential for integrating topological features with other features in the subsequent work, we used the DE feature as the key complementary feature. DE is an entropy-based metric that excels at capturing the nonlinear information complexity of emotional EEG, it is calculated by the following equation:

(1) $$h(X)=-\int_{\;X}f(x)\log(f(x))dx$$

where f(x) is the probability density function of x. If the EEG signal X used is a time series obeying a Gaussian distribution $N(\mu ,\sigma^{2})$, then its differential entropy is

(2) $$h(X)=-\int_{-\infty }^{\infty }\frac{1}{\sqrt{2\pi \sigma^{2}}}e^{-\frac{x-\mu }{2\sigma^{2}}}\log(\frac{1}{\sqrt{2\pi \sigma^{2}}}e^{-\frac{x-\mu }{2\sigma^{2}}})dx=\frac{1}{2}\log(2\pi e\sigma^{2})$$

### 4.3. Parameters Determination

**Figure 2:**
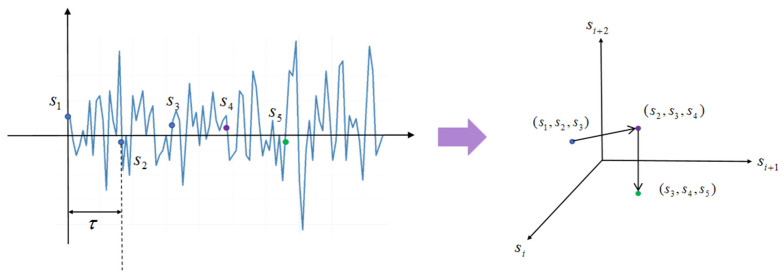
Schematic diagram of the reconstruction process. The points $s_{1},\;s_{2},\;s_{3},\;s_{4},\;s_{5}$ were in the original sequence. After the reconstruction of τ = 1 and d = 3, three consecutive points $\left(s_{1},s_{2},s_{3}\right),\;\left(s_{2},s_{3},s_{4}\right),\;(s_{3},s_{4},s_{5})$ are obtained in the new space and form a continuous trajectory.

For the phase space reconstruction process, if $s_{n}$ is the nth point in the raw EEG sequence, the points in the space are reconstructed in the following way:

(3) $$P_{n}=(s_{n},s_{n+\tau },\ldots ,s_{n+(d-1)\tau })$$

where $P_{n}$ represents the nth point in the phase space after reconstruction. τ and d are time delay and the embedding dimension. Figure 2 shows a schematic diagram of the reconstruction process.

In this paper, we adopt the minimum cross prediction error (MCPE) method to determine τ, which is based on the polynomial regression model to minimize prediction error and is able to adaptively capture complex dynamics and suppress noise interference. At the same time, we utilize the Grassberger–Procaccia (G–P) method to compute d, which dynamically quantifies the geometric characteristics of trajectories through the proximity density of pairs of points in phase space in order to preserve the topology of the system, balancing low computational complexity with the ability to analyze dynamic properties.

#### 4.3.1. Determination of Time Delay—Minimum Cross Prediction Error (MCPE)

**Figure 3:**
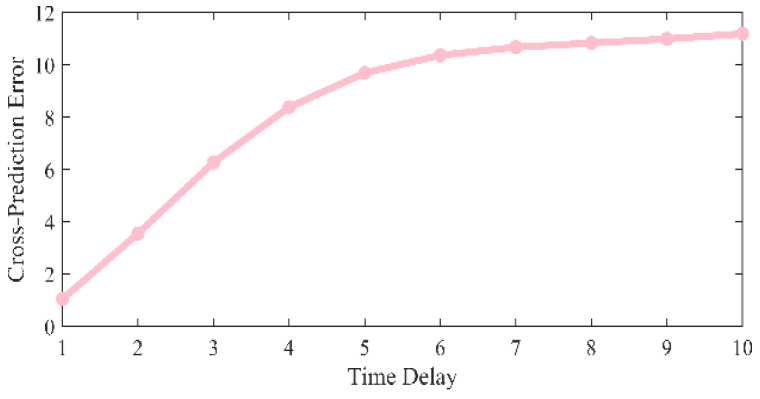
Comparison of MSE at different time delays.

The core idea of MCPE is to determine the time delay by predicting the future values of the time series $T\;=\;(t_{1},{\;t}_{2},\;t_{3},\;\ldots ,\;t_{n})$, and subsequently, for different time delay parameters, the original data is shifted cyclically to obtain the sequence $Z\;=\;(z_{1},{\;z}_{2},{\;z}_{3},\;\ldots ,\;z_{n})$ in order to simulate the effect of time delay. A quadratic polynomial regression was utilized to fit the data with the fitting equation

(4) $$T=q_{0}+q_{1}Z+q_{2}Z^{2}$$

where $q_{0},\;q_{1},\;\mathrm{and}\;q_{2}$ are the constant, first-order, and second-order coefficients. Through regression analysis, we estimate the optimal parameter $q_{0},{\;q}_{1},\;q_{2}$. These estimated values are substituted into (3) to calculate the predicted values ${\;T}_{\mathrm{pred}}$:

(5) $$T_{pred}=\hat{q_{0}}+\hat{q_{1}}Z+\hat{q_{2}}Z^{2}$$

further calculate the mean square error (MSE) between the predicted value and the actual value:

(6) $$\mathrm{MSE}=\frac{1}{n}\sum_{i=1}^{n}(T_{i}-T_{pred,i}{)}^{2}$$

where the comparison of the MSE corresponding to different time delays is shown in Figure 3. When τ = 1, the minimum MSE is obtained.

#### 4.3.2. Determination of Embedding Dimension—Grassberger–Procaccia (G–P) Method

**Figure 4:**
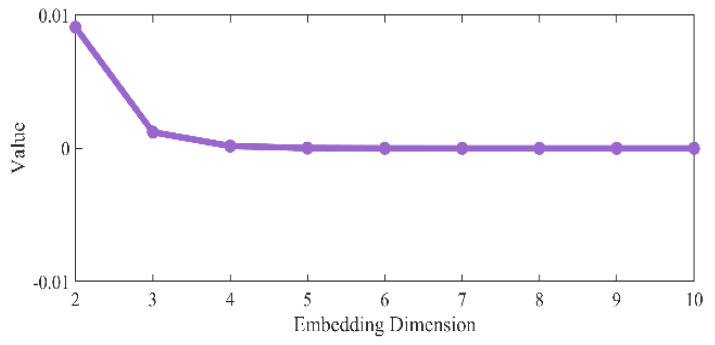
Comparison of $C_{m}$ values for different embedding dimensions.

The G–P method aims to determine the lowest embedding dimension that can truly reflect the dynamic characteristics of the system by analyzing the point-to-point distance relationship in the phase space. The specific process is as follows: based on the time delay parameter that has been determined by MCPE, the phase space under different embedding dimensions is reconstructed from 2 dimensions to 10 dimensions. For the reconstructed phase space in each dimension, calculate the number of point pairs $C_{t}$, count the distance $L_{t}$ between two points in each point pair, and then filter out the point pairs $C_{s}$ whose distance $L_{t}$ is less than the standard deviation of the original time series. We used the scale value

(7) $$C_{m}=\frac{C_{s}}{C_{t}}$$

to compare the values of $C_{m}$ under different embedding dimensions, as shown in Figure 4, and found that the value of $C_{m}$ gradually tends to stabilize from three dimensions. This indicates that the 3-dimensional phase space can effectively characterize the dynamics of the system.

### 4.4. Dimension Reduction of Trajectories

**Figure 5:**
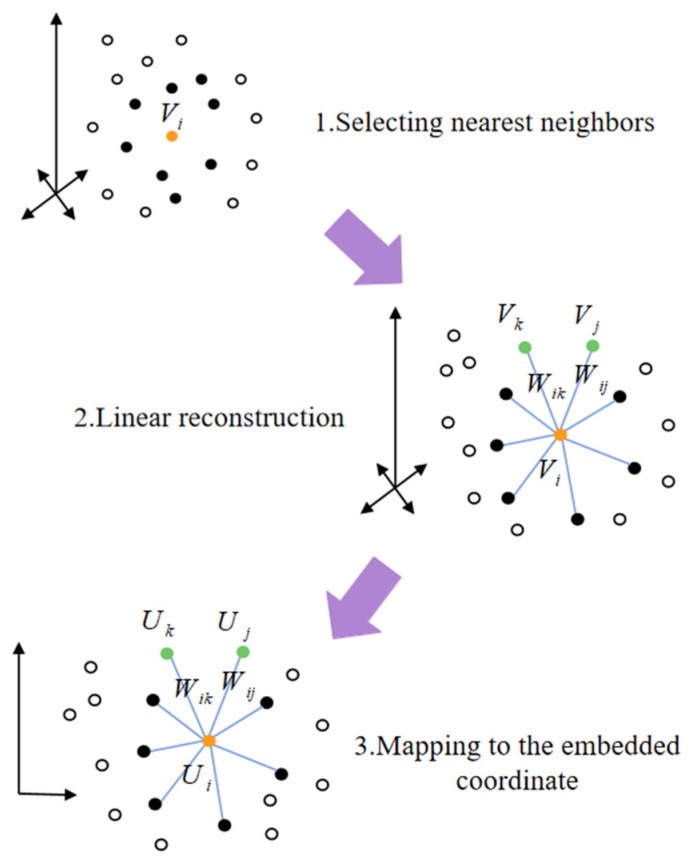
The main process LLE.

After obtaining the high-dimensional trajectories, downscaling the trajectories to a 2D plane enables better visualization and subsequent feature construction. It is also necessary to preserve important similarities and relationships while maintaining the local structure of the original high-dimensional data, thus enhancing the effectiveness of the analysis. We utilize local linear embedding (LLE) for dimensionality reduction, which is able to effectively preserve the local structure of the data by assuming that the data is linear to the local neighborhood and maintaining the relative positions of the data points in their local neighbors unchanged [35]. Figure 5 shows a schematic diagram, which is realized in the following stages:

For each point $V_{1},{\;V}_{2},\;\ldots ,{\;V}_{n}$ in the high-dimensional space, calculate its Euclidean distance from the other points around it and select the p points with the closest distance as neighbors, denoted as $\left\{V_{i_{1}}{,\;V}_{i_{2}},\;\ldots ,{\;V}_{i_{p}}\right\}$.

Subsequently, a local linear weight matrix W is determined by minimizing the reconstruction error. It is assumed that each sample point can be linearly reconstructed by its neighbors, and the weight $W_{ij}$ denotes the contribution of point $V_{i}$ to the jth neighbor. The minimization objective of the reconstruction error (RE) is defined as

(8) $$\mathrm{RE}=\min\sum_{i=1}^{n}||V_{i}-\sum_{j=1}^{p}W_{ij}V_{i_{j}}{\left|\right|}^{2}$$

meanwhile, it is required that the sum of the weights of each sample point must be equal to 1. The sparse weight matrix W is obtained by the solution.

Finally, in the low-dimensional space embedding stage, the weight matrix is utilized to construct the symmetric matrix as

(9) $$M={\left(I-W\right)}^{T}(I-W)$$

where I is the unit matrix. The eigenvalue recomposited by

(10) Mμ=λμ

Select the eigenvectors $\mu_{2},\;\mu_{3}$ corresponding to the first two smallest eigenvalues, except the smallest eigenvalue $\mu_{1},$ and arrange them to generate the low-dimensional embedding U:

(11) $$U={\left[\mu_{2},\mu_{3}\right]}^{T}$$

**Figure 6:**
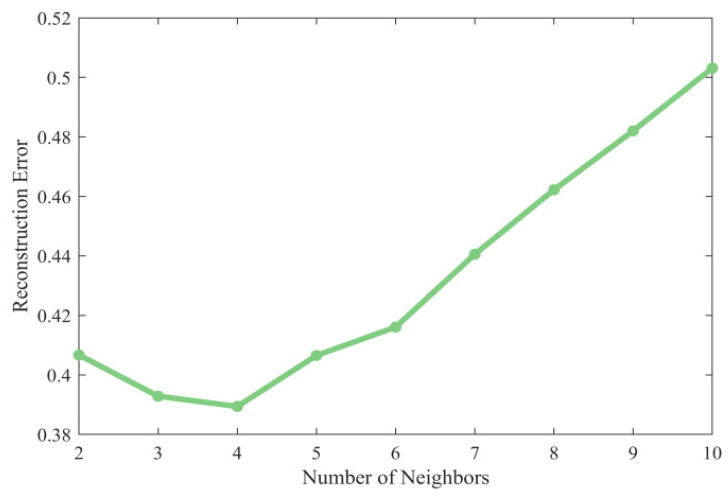
Comparison of reconstruction error (RE) with different number of nearest neighbors (the number of nearest neighbors should not be smaller than the dimension after dimensionality reduction, so there is no p = 1 for the horizontal coordinate).

In LLE, the selection of the number of nearest neighbors p has a significant effect on the dimensionality reduction effect. Based on the principle of minimizing the reconstruction error described in the previous section, the optimal p value needs to be determined by systematic parameter selection. We calculated the RE at different p values, as shown in Figure 6, and found that the RE reaches the global minimum when p = 4.

### 4.5. Construction of Topological Features

**Figure 7:**
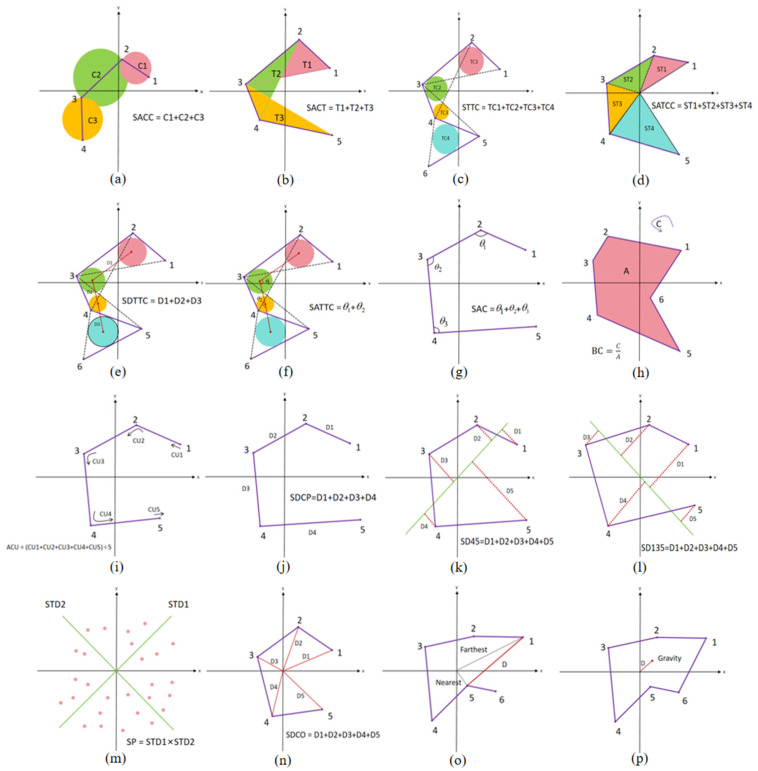
Schematic diagram of topological features. (**a**) SACC; (**b**) SACT; (**c**) STTC; (**d**) SATTC; (**e**) SDTTC; (**f**) SATCC; (**g**) SAC; (**h**) BC; (**i**) ACU; (**j**) SDCP; (**k**) SD45; (**l**) SD135; (**m**) SP; (**n**) SDCO; (**o**) DBNF; (**p**) DGCO.

Downscaling the high-dimensional trajectories by LLE, the trajectories in the 2D plane under different emotions can be obtained. We designed 16 topological features based on the differences among trajectories under different emotions. These features are quantified from three perspectives: the trajectory range area, the degree of trajectory edge distortion, and the distance of trajectory points from the coordinate center. A schematic of all the features is shown in Figure 7.

#### 4.5.1. Area of the Trajectory

Such features characterize the spatial dynamic range of neural oscillations in different emotions by quantifying the difference in the area of the region covered by the trajectory. The significant difference in the degree of diffusion of phase space trajectories aimed at capturing high arousal emotions versus low arousal emotions is directly related to the nonlinear dynamic properties of the EEG signals.

***(a) Sum of the area of consecutive circles (SACC):*** Every two consecutive points form a vector. Consider the length of the vector as the diameter of the circle, find the area of the circle, and calculate the sum of the areas of all the circles:

(12) $$\mathrm{SACC}=\sum_{i=1}^{N-1}\frac{\pi }{4}[{\left(X_{i+1}-X_{i}\right)}^{2}+{\left(Y_{i+1}-Y_{i}\right)}^{2}]$$

where N is the number of points in the 2-dimensional plane, $X_{i}\;\mathrm{and}\;Y_{i}$ are the horizontal and vertical coordinates of the points on the plane.

***(b) Sum of the area of consecutive triangles (SACT):*** For every three consecutive points that form a triangle, find the area of that triangle and calculate the sum of the areas of all the triangles:

(13) $$\mathrm{SACT}=\frac{1}{2}\sum_{i=1}^{N-2}\left|\det\left[\begin{array}{ccc} X_{i} & Y_{i} & 1\\ X_{i+1} & Y_{i+1} & 1\\ X_{i+2} & Y_{i+2} & 1 \end{array}\right]\right|$$

***(c) Sum of the area of triangles’ tangent circles (STTC):*** Every three consecutive points form a triangle. Find the incircle of all the triangles and calculate the sum of the areas. The three sides a, b, and c of the triangle are calculated as follows:

(14) $$a=\sqrt{{\left(X_{i+1}-X_{i}\right)}^{2}+{\left(Y_{i+1}-Y_{i}\right)}^{2}}$$

(15) $$b=\sqrt{{\left(X_{i+2}-X_{i+1}\right)}^{2}+{\left(Y_{i+2}-Y_{i+1}\right)}^{2}}$$

(16) $$c=\sqrt{{\left(X_{i+2}-X_{i}\right)}^{2}+{\left(Y_{i+2}-Y_{i}\right)}^{2}}$$

the area S of the triangle is calculated as follows:

(17) $$S=\sqrt{\frac{a+b+c}{2}\times \frac{b+c-a}{2}\times \frac{a+c-b}{2}\times \frac{a+b-c}{2}}$$

the radius r of its internal tangent circle is calculated as follows:

(18) $$r=\frac{2S}{a+b+c}$$

the final result of the STTC calculation is obtained as follows:

(19) $$\mathrm{STTC}=\sum_{j=1}^{N-2}\pi r_{j}^{2}$$

***(d) Sum of the area of the triangles formed by consecutive points and the coordinate origin (SATCC):*** Every two consecutive points and the origin of the coordinate center form a triangle and the sum of the areas of all the triangles is calculated. Then the three sides α, β, and γ of the triangle they form are calculated as

(20) $$\alpha =\sqrt{X_{i}^{2}+Y_{i}^{2}}$$

(21) $$\beta =\sqrt{X_{i+1}^{\;\;\;\;2}+Y_{i+1}^{\;\;\;\;2}}$$

(22) $$\gamma =\sqrt{{\left(X_{i+1}-X_{i}\right)}^{2}+{\left(Y_{i+1}-Y_{i}\right)}^{2}}$$

calculate the area $S_{c}$ of the triangle using Helen’s formula:

(23) $$S_{c}=\sqrt{\frac{\alpha +\beta +\gamma }{2}\times \frac{\beta +\gamma -\alpha }{2}\times \frac{\alpha +\gamma -\beta }{2}\times \frac{\alpha +\beta -\gamma }{2}}$$

the final result of the SATCC calculation is obtained as

(24) $$\mathrm{SATCC}=\sum_{j=1}^{N=2}S_{c}$$

***(e) Sum of the distance from the centers of the triangle tangent circles (SDTTC):*** After finding the tangent circles of the triangle according to the method in (c), the sum of the distance between the centers of all the tangent circles is calculated. The horizontal and vertical coordinates $X_{c}$ and $Y_{c}$ of the centers of the tangent circles of the triangle are calculated as follows:

(25) $$X_{c}=\frac{X_{i}+X_{i+1}+X_{i+2}}{3}$$

(26) $$Y_{c}=\frac{Y_{i}+Y_{i+1}+Y_{i+2}}{3}$$

Two sequences can be obtained for the horizontal and vertical coordinates of the center of the circle:

(27) $$X(c)=[X_{c_{1}},X_{c_{2}},X_{c_{3}},\ldots ,X_{c_{n-2}}]$$

(28) $$Y(c)=[Y_{c_{1}},Y_{c_{2}},Y_{c_{3}},\ldots ,Y_{c_{N-2}}]$$

then SDTTC can be calculated as

(29) $$\mathrm{SDTTC}=\sum_{k=1}^{N-3}\sqrt{{\left(X_{C_{k+1}}-X_{C_{k}}\right)}^{2}+{\left(Y_{C_{k+1}}-Y_{C_{k}}\right)}^{2}}$$

***(f) Sum of the area of the triangles formed by consecutive points and the coordinate origin (SATCC):*** After finding the tangent circles of the triangle according to the method in (c), the centers of the tangent circles are connected to form continuous vectors and the sum of the angle degrees between these continuous vectors is calculated. Based on the sequence of horizontal and vertical coordinates of the circle centers calculated in (e), we can calculate the SATTC as

(30) $$\mathrm{SATTC}=\sum_{k=1}^{N-4}\frac{\left(X_{C_{k+1}}-X_{C_{k}})(X_{C_{k+2}}-X_{C_{k+1}})+(Y_{C_{k+1}}-Y_{C_{k}})(Y_{C_{k+2}}-Y_{C_{k+1}}\right)}{\sqrt{{\left(X_{C_{k+1}}-X_{C_{k}}\right)}^{2}+{\left(Y_{C_{k+1}}-Y_{C_{k}}\right)}^{2}}+\sqrt{{\left(X_{C_{k+2}}-X_{C_{k+1}}\right)}^{2}+{\left(Y_{C_{k+2}}-Y_{C_{k+1}}\right)}^{2}}}$$

#### 4.5.2. Degree of Distortion of Trajectory Edge

Such features quantify the mutation characteristics of emotion-induced neural activities by analyzing the local curvature, angle, and boundary complexity of the trajectory path. These features can effectively distinguish high-frequency nonlinear changes in emotional fluctuations and make up for the inadequacy of traditional linear features in capturing dynamic mutations.

***(g) Sum of the angle between consecutive vectors (SAC):*** Vectors are formed between every two consecutive points and angles are formed between every two consecutive vectors. Calculate the sum of the degrees of all the angles as follows:

(31) $$\mathrm{SAC}=\sum_{i=1}^{N-2}\frac{\left(X_{i+1}-X_{i})(X_{i+2}-X_{i+1})+(Y_{i+1}-Y_{i})(Y_{i+2}-Y_{i+1}\right)}{\sqrt{{\left(X_{i+1}-X_{i}\right)}^{2}+{\left(Y_{i+1}-Y_{i}\right)}^{2}}+\sqrt{{\left(X_{i+2}-X_{i+1}\right)}^{2}+{\left(Y_{i+2}-Y_{i+1}\right)}^{2}}}$$

***(h) Boundary complexity (BC):*** Calculate the perimeter of the boundary and the area of the region enclosed by the boundary for all points, and calculate the ratio of the perimeter to the area.

Using the convex packet algorithm to obtain the outermost boundary point, join its head and tail together and form a closed-loop region, calculate the perimeter C of the region boundary, and calculate the area A enclosed by the region:

(32) $$\mathrm{BC}=\frac{C}{A}$$

***(i) Average curvature (ACU):*** In the trajectory formed by consecutive points, the curvature at each point is calculated and averaged. Assuming that U and V represent two continuous vectors. The curvature CU is computed as

(33) $$\mathrm{CU}=\frac{2\times |U\times V|}{\left|U|\times |V|\times |U\times V\right|}$$

the average curvature ACU is calculated as

(34) $$\mathrm{ACU}=\frac{1}{N}\sum_{i=1}^{N}CU_{i}$$

For the curvature at the first and the last point in the trajectory, it is assigned to the curvature of the neighboring points: ${\mathrm{CU}}_{1}\;=\;{\mathrm{CU}}_{2}$, ${\mathrm{CU}}_{N}\;=\;{\mathrm{CU}}_{N-1}$.

***(j) Sum of the distance between consecutive points (SDCP):*** Two consecutive points are constructed into a vector and the sum of the lengths of all vectors is calculated:

(35) $$\mathrm{SDCP}=\sum_{i=1}^{N-1}\sqrt{{\left(X_{i+1}-X_{1}\right)}^{2}+{\left(Y_{i+1}-Y_{1}\right)}^{2}}$$

#### 4.5.3. Distance of the Trajectory Point from the Coordinate Center

Such features explain the spatial distribution pattern of neural activity in emotional processing by calculating the relationship between the distance of a trajectory point and the coordinate origin.

***(k) Sum of the distance of each point from the 45-degree line (SD45):*** Calculate the sum of the distances between all points and the 45-degree angle bisector of the coordinate system:

(36) $$\mathrm{SD}45=\sum_{i=1}^{N}\frac{\left|Y_{i}-X_{i}\right|}{\sqrt{2}}$$

***(l) Sum of the distance of each point from the 135-degree line (SD135):*** Calculate the sum of the distances between all points and the 135-degree angle bisector of the coordinate system:

(37) $$\mathrm{SD}135=\sum_{i=1}^{N}\frac{\left|X_{i}+Y_{i}\right|}{\sqrt{2}}$$

***(m) Scatter for all points (SP):*** Calculate the distance of each point from the 45-degree angle bisector $d_{45}$ and the distance from the 135-degree angle bisector $d_{135}$:

(38) $$d_{45(i)}=\frac{\left|Y_{i}-X_{i}\right|}{\sqrt{2}}$$

(39) $$d_{135(i)}=\frac{\left|X_{i}+Y_{i}\right|}{\sqrt{2}}$$

next the standard deviation is calculated in each of the two directions:

(40) $$\mathrm{STD}45=std[d_{45}(1),d_{45}(2),d_{45}(3),\ldots ,d_{45}(n)]$$

(41) $$\mathrm{STD}135=std[d_{135}(1),d_{135}(2),d_{135}(3),\ldots ,d_{135}(n)]$$

calculating scatter for all points:

(42) SP=STD45×STD135

***(n) Sum of the distance to coordinate origin (SDCO):*** Calculate the sum of the distances between all points and the origin of the coordinate system:

(43) $$\mathrm{SDCO}=\sum_{i=1}^{N}\sqrt{X_{i}^{\;2}+Y_{i}^{2}}$$

***(o) Distance between the nearest point and the farthest point from the coordinate origin (DBNF):*** Among all the points, find out the nearest point $\left(X_{\mathrm{near}},\;Y_{\mathrm{near}}\right)$ and the farthest point $\left(X_{\mathrm{far}},\;Y_{\mathrm{far}}\right)$ from the origin by the comparison method and calculate the distance between these two points:

(44) $$\mathrm{DBNF}=\sqrt{{\left(X_{\mathrm{far}}-X_{\mathrm{near}}\right)}^{2}+{\left(Y_{\mathrm{far}}-Y_{\mathrm{near}}\right)}^{2}}$$

***(p) Distance from the gravity center to the origin (DGCO):*** Calculate the center of gravity of all points and calculate the distance from the center of gravity to the origin of the coordinates. $X_{g}$ and $Y_{g}$ are the horizontal and vertical coordinates of the center of gravity, respectively, and they are computed by calculating the mean of the coordinates of the corresponding dimension as follows:

(45) $$X_{g}=\frac{1}{N}\sum_{i=1}^{N}X_{i}$$

(46) $$Y_{g}=\frac{1}{N}\sum_{i=1}^{N}Y_{i}$$

(47) $$\mathrm{DGCO}=\sqrt{X_{g}^{\;2}+Y_{g}^{2}}$$

## 5. Results

In this paper, seven traditional classifiers, linear discriminant analysis (LDA), Gaussian Naive Bayes (GNB), K-nearest neighbor (KNN), support vector machine (SVM), random forest (RF), extreme gradient boosting (XGBoost), and convolutional neural network (CNN) were selected and the parameter settings of each classifier are shown in Table 2. For the SEED, we conduct subject-dependent experiments for 2-Class (happy and sad) and 3-Class (happy, neutral, and sad). For the HIED, there were three subject-dependent experiments for 2-Class (happy and sad), 3-Class (happy, neutral, and sad), and 4-Class (happy, neutral, sad, and afraid). For each experiment, we utilized the first 80% of the fragment samples as the training set and the last 20% of the fragment samples as the test set.

Table 3 shows the classification performance on the SEED and HIED datasets. The results indicate that the XGBoost classifier achieves the best performance on the SEED dataset for both 2-Class and 3-Class tasks. Additionally, for the HIED dataset, the XGBoost classifier excels in 3-Class and 4-Class tasks. It is worth noting that CNN, as one of the most basic deep learning models, did not show significantly better performance than the tree model in our experiments, and only slightly outperformed the XGBoost classifier for the 2-Class task on the HIED, which may be due to the tree model’s superior ability to cope with irregularities and nonlinearities in tabular data [36].

**Figure 8:**
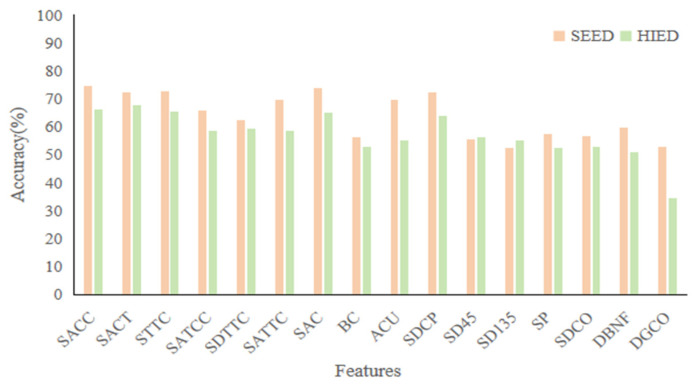
Results of 16 topological features in the case of separate experiments.

We analyzed the results for each topological feature separately in both datasets, as shown in Figure 8. In the SEED, which was evaluated using the 3-Class task, the best performing feature was SACC with an accuracy of 74.44%. In terms of overall performance, the five features SACC, SACT, STTC, SAC, and SDCP in the SEED all have accuracy rates above 70%. In the HIED, evaluated using the 4-Class task, the best performing feature was SACT with an accuracy of 67.6%, and the features SACC, SACT, STTC, SAC, and SDCP also have better performance than other features, with accuracy rates above 60%. This indicates that these five features show stable performance in the classification task under different datasets, suggesting that they have universal characterization ability for emotion recognition.

**Figure 9:**
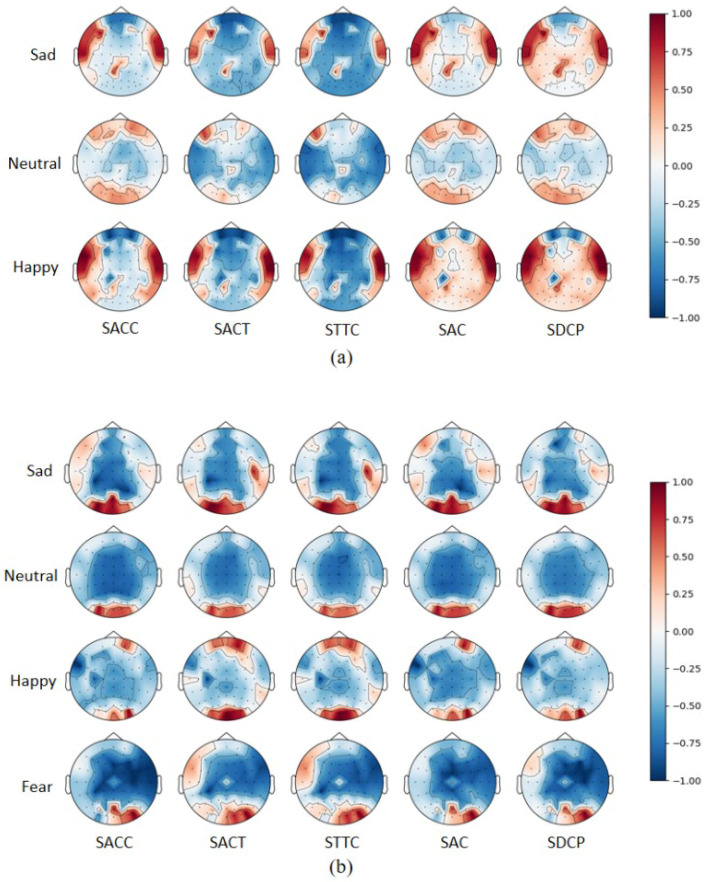
Mean brain topographies of five topological features in different emotions: (**a**) is for normal-hearing subjects in the SEED and (**b**) is for hearing-impaired subjects in the HIED.

For electrode channel selection, we first plotted and analyzed brain topography maps of the five selected topological features based on the normalized post-averaged samples of all normal-hearing and hearing-impaired subjects under different emotions. As shown in Figure 9, in normal-hearing subjects, significant brain regions for neutral emotions were concentrated in the frontal and occipital lobes, and sad and happy emotions produced different levels of activation in the temporal lobes compared to neutral emotions. In the subjects with hearing impairments, the activation of occipital lobe regions was more pronounced, while activity in the temporal lobe was notably reduced. This phenomenon is likely linked to the brain’s visual compensation mechanism, where the lack of auditory input leads to enhanced visual and somatosensory pathways. Through cross-modal neural reorganization, resources are optimized, resulting in greater efficiency of the occipital lobe in processing emotion-related visual cues.

**Figure 10:**
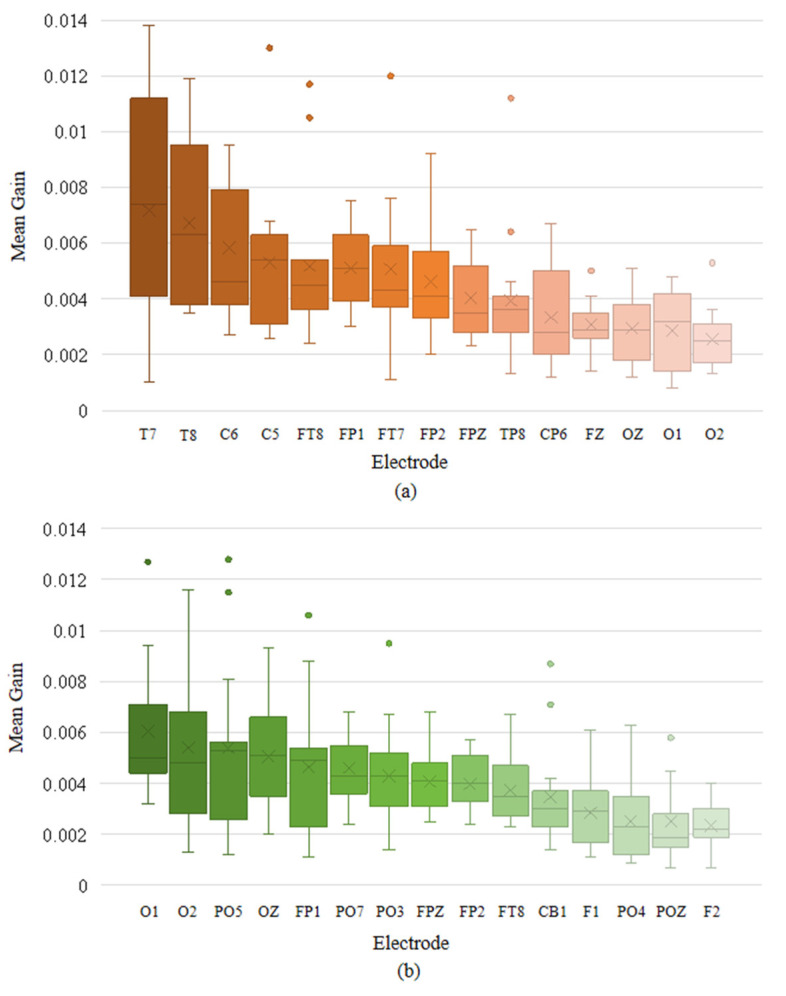
Top-ranked electrodes by mean XGBoost gain in descending order: (**a**) for normal-hearing subjects in the SEED and (**b**) for hearing-impaired subjects in the HIED.

**Figure 11:**
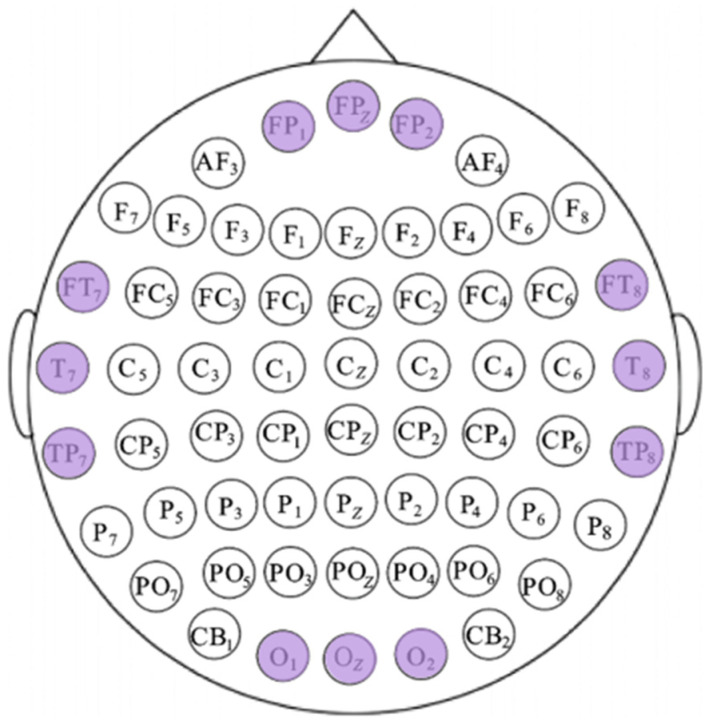
Schematic diagram of electrode selection, wherein the purple electrode is selected.

We also calculated the mean XGBoost gain for various features at each electrode channel and plotted the box plots of the top-ranked channels with the highest mean gain in descending order, as illustrated in Figure 10. In normal-hearing subjects, the electrodes with higher gain are mainly concentrated in the temporal lobe region (T7, T8, C6, C5, FT8, FT7, TP8, and CP6). Additionally, the electrodes in the parietal lobe (FP1, FP2, FPZ, and FZ) and the occipital lobe (OZ, O1, and O2) also have relatively prominent contributions. In hearing-impaired subjects, the high-contributing electrodes in the occipital lobe are significantly more numerous compared to normal-hearing subjects (O1, O2, PO5, OZ, PO7, PO3, CB1, PO4, and POZ), while the temporal lobe only has FT8. This indicates that the emotional classification of hearing-impaired subjects is more dependent on the occipital lobe, which is consistent with the previous statement. The other electrodes with high contributions are mainly located in the parietal lobe (FP1, FPZ, FP2, F1, and F2). Based on the above information, we combined the key brain region distributions of the two groups of subjects, taking into account both the common areas and the bilateral symmetry, and selected 12 high-gain electrodes, as shown in Figure 11.

The emotion recognition results were carried out based on the optimal five topological features and we selected 12 EEG electrodes in the two datasets, as shown in Table 4. It can be seen that the recognition accuracy of most subjects improved to varying extents following feature selection and electrode selection. In the SEED, the average accuracy increased by 3.17% for the 2-Class task and 6.44% for the 3-Class task. In the HIED, the average accuracy of the 2/3/4-Class tasks increased by 3.17%/3%/4.26%, respectively.

## 6. Discussion

### 6.1. Analysis of Topological Feature Differences and Cross-Group Classification Performance

In order to further explore the similarities and differences in neural representation mechanisms between normal-hearing and hearing-impaired subjects with the five selected topological features (SACC, SACT, STTC, SAC, and SDCP), quantitative analyses were carried out, as shown in Table 5 and Table 6.

In within-group analyses, the five topological features showed similar trends for both normal-hearing and hearing-impaired subjects: neutral emotions consistently showed the smallest values across all features, while happy emotions showed the highest. This pattern suggests that neutral emotional states are associated with more stable and less variable neural dynamics, whereas happy emotions elicit broader and more complex neural oscillations.

In inter-group analyses, the mean values of all five features were generally higher in hearing-impaired subjects compared to normal-hearing subjects across all emotional states. This indicated that the trajectories of EEG signals in hearing-impaired subjects cover a wider spatial range and exhibit more complex local structures, which may reflect adaptive neural mechanisms in response to auditory deprivation.

**Figure 12:**
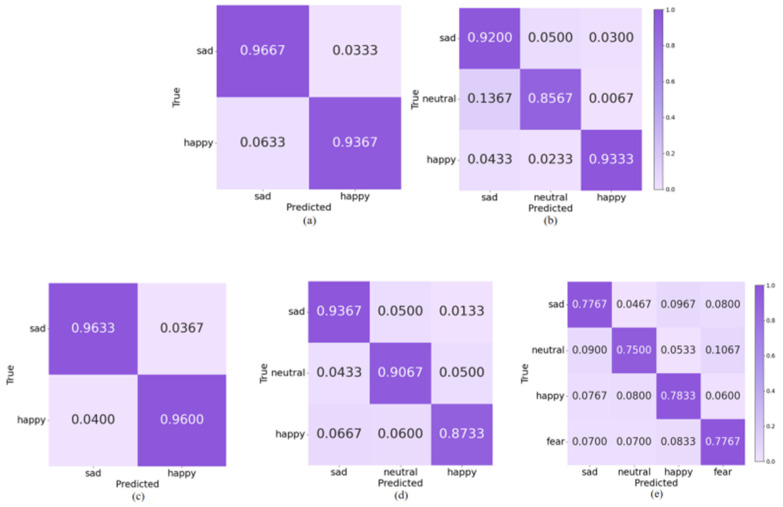
Classification confusion matrix based on topological features: (**a**) 2-Class in SEED, (**b**) 3-Class in SEED, (**c**) 2-Class in HIED, (**d**) 3-Class in HIED, (**e**) 4-Class in HIED.

In order to explore the performance of topological features in recognizing different emotions, we plotted the average confusion matrix of all subjects in the different emotion categorization tasks mentioned above. As shown in Figure 12, in the SEED, the accuracy rate of sad emotions in the 2-Class task was higher than that of happy emotions; in the 3-Class task, the accuracy rate of both sad and happy emotions was more than 90%, and that of the neutral emotions was lower, with 13.67% of the samples were incorrectly predicted to be sad, which demonstrates that although topological features are effective in amplifying the variability of different emotions among normal-hearing subjects, there may still be some overlap in the nonlinear dynamic representation of low arousal states. In the HIED, the recognition accuracies of both emotions under the 2-Class task exceeded 95%; the recognition rate of happy emotions in the 3-Class task was significantly lower than that of sad and neutral; and the best performing emotion for recognition in the 4-Class task was happy with 78.33%, which suggests that the topological features can more effectively capture the synergistic dynamic properties of happy emotions in hearing-impaired subjects.

The comparison of our experimental results with previous studies on the SEED is presented in Table 7, which shows the superiority of the topological features we used. Compared with the limitations of other features, our topological features can better quantify the nonlinear dynamic patterns of brain electrical activity related to emotions.

To explore whether topological features have the potential to combine with other features to improve emotion recognition accuracy, we conducted a fusion experiment by combining our topological features with DE. As extended in Table 7, the overall average accuracy of the fused “Topological Features + DE” scheme reached 92.50% on the SEED 3-Class task—2.17% higher than topological features alone (90.33%). This improvement confirms the complementary value between the two: topological features lay the foundation for capturing the spatial dynamic patterns of emotional EEG, while DE supplements critical frequency–domain information to enhance performance.

### 6.2. Cross-Group 6-Class Task

**Figure 13:**
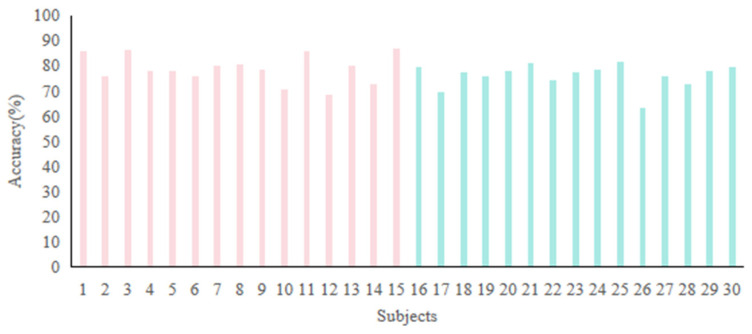
Recognition accuracies of cross-group 6-Class task: subjects 1 to 15 are normal-hearing people in the SEED, while subjects 16 to 30 are hearing-impaired people in the HIED.

To enhance the generalizability of emotion recognition systems across diverse people, we merged the SEED and HIED into a unified dataset comprising 30 subjects, implementing leave-one-subject-out validation for a cross-group 6-Class task (2 groups × 3 emotions: sad, neutral, and happy). Figure 13 shows the accuracies. The majority of subjects achieved recognition accuracies above 70%, with the highest accuracy of 86.67% observed in subject 15. Subject 26 exhibited the lowest accuracy of 63.33%, which may be attributed to individual variability in neurophysiological responses. The average accuracy across all subjects was 77.5%, demonstrating the robust generalization capability of the proposed method.

**Figure 14:**
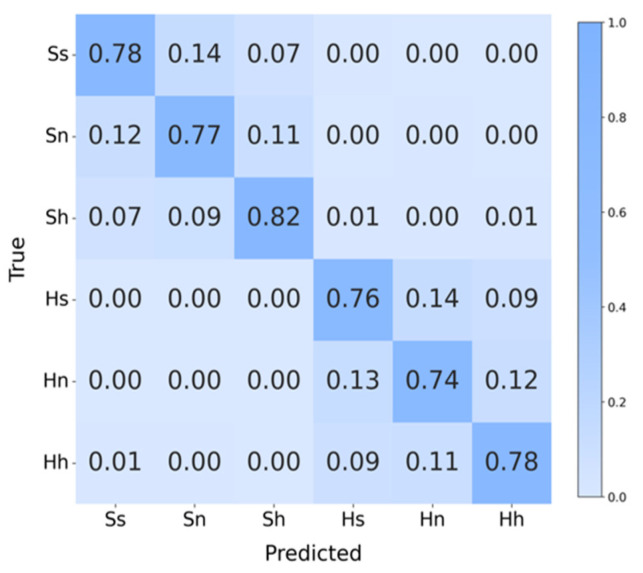
Confusion matrix of cross-group 6-Class task. Ss (SEED-sad), Sn (SEED-neutral), Sh (SEED-happy), Hs (HIED-sad), Hn (HIED-neutral), Hh (HIED-happy).

Figure 14 is the confusion matrix of the task, it can be seen that the highest recognition accuracy is for happy in normal-hearing subjects with 82%, while the lowest is for sad in hearing-impaired subjects with 74%. In addition, only a very small number of samples showed cross-group classification errors, which indicates that the topological features can quantify the emotional differences between the two groups of subjects very well, and further confirms that the features capture emotions’ common dynamic patterns across groups, verifying the method’s strong generalization.

## 7. Conclusions

In this paper, we convert EEG signals into spatial trajectories using phase space reconstruction, and construct 16 different topological features based on the differences in trajectories in different emotions. After obtaining the preliminary results, we obtained more meaningful features and important brain regions for feature and electrode selection based on experiments with independent features as well as brain topographic mapping, thus reducing the model’s computation time and increased the recognition accuracy. Next, we analyzed the relationship between the size of the average values of the five high-quality topological features in different groups. In the between-group comparison, we found that hearing-impaired subjects had significantly larger values in the five features than normal-hearing subjects; in the within-group comparison, we found that there was a clear trend in the values of the features in different emotions: neutral < negative < positive, for both normal-hearing and hearing-impaired subjects. The average accuracy of the 2/3-Class task in the SEED was 95.16%/90.33%. The average accuracy of 2/3/4-Class task in the HIED was 96.16%/90.89%/77.17%. In addition, to build more compatible emotion recognition techniques, we conducted a cross-group 6-Class task and obtained an average classification accuracy of 77.5%. In future work, we plan to use phase space images in combination with complementary manually extracted features to further improve the generalization of the emotion recognition algorithm.

## Figures and Tables

**Table 1:** Movie clip details.

No.	Titles	Labels	Start Time Point	Duration (s)
1	Lost in Thailand	Happy	0:06:13	238
2	Coming Soon	Fear	0:01:57	192
3	World Heritage in China-02	Neutral	0:00:50	226
4	Aftershock	Sad	0:20:10	205
5	Back to 1942	Sad	0:49:58	242
6	Coming Soon	Fear	1:09:13	189
7	World Heritage in China-02	Neutral	0:10:40	221
8	Lost in Thailand	Happy	1:05:10	204
9	Flirting Scholar	Happy	1:18:57	266
10	World Heritage in China-13	Neutral	0:02:59	184
11	Back to 1942	Sad	2:01:21	239
12	Dead Silence	Fear	0:54:50	211
13	Dead Silence	Fear	1:15:47	182
14	World Heritage in China-13	Neutral	0:10:41	240
15	A Chinese Odyssey Part 1-Pandora’s Box	Happy	0:11:32	241
16	Back to 1942	Sad	2:16:37	240
17	After shock	Sad	1:48:53	205
18	The Conjuring	Fear	1:17:42	200
19	World Heritage in China-21	Neutral	0:05:36	240
20	A Chinese Odyssey Part 1-Pandora’s Box	Happy	0:35:00	242

**Table 2:** Parameter settings for each classifier.

Classifier	Parameter Setting
LDA	solver = ‘svd’, n_components = 1
GNB	-
KNN	n_neighbors = 5
SVM	kernel = linear, C = 10, gamma = 200
RF	n_estimators = 200, max_depth = 10, min_samples_split = 3, min_samples_leaf = 1
XGBoost 2.1.1.	n_estimators = 100, max_depth = 10, learning_rate = 0.1
CNN	epoch = 50, learning rate = 0.001, optimizer: Adam, batch_size = 32

**Table 3:** Classification results under different classifiers (%).

Classifier	SEED		HIED		
	2-Class (Happy/Sad)	3-Class (Happy/Neutral/Sad)	2-Class (Happy/Sad)	3-Class (Happy/Neutral/Sad)	4-Class (Happy/Neutral/Sad/Afraid)
LDA	67.50	46.67	85.00	80.33	53.17
GNB	65.50	47.78	77.00	63.00	46.50
KNN	75.83	64.44	86.67	84.00	64.08
SVM	80.17	72.11	91.67	86.56	68.25
RF	89.17	80.33	93.67	86.89	69.31
XGBoost	92.00	83.88	93.00	87.88	72.92
CNN	85.00	74.44	93.66	87.11	70.65

**Table 4:** Comparison of accuracy per subject with and without feature and EEG electrode channel selection (%).

Subject	SEED				HIED					
	2-Class Without Selection	2-Class with Selection	3-Class Without Selection	3-Class with Selection	2-Class Without Selection	2-Class with Selection	3-Class Without Selection	3-Class with Selection	4-Class Without Selection	4-Class with Selection
1	90	92.5	81.67	86.67	95	100	86.67	91.67	77.5	81.25
2	90	97.5	73.33	83.33	95	95	86.67	90	71.25	72.5
3	90	90	81.67	91.67	87.5	92.5	88.33	91.67	76.25	78.75
4	82.5	95	83.33	88.33	97.5	100	91.67	93.33	76.25	82.5
5	92.5	85	86.67	81.67	92.5	97.5	90	96.67	67.5	78.75
6	87.5	90	86.67	91.67	87.5	92.5	83.33	90	77.5	83.75
7	87.5	100	88.33	96.67	92.5	95	98.33	90	68.75	77.5
8	97.5	97.5	80	83.33	90	92.5	81.67	88.33	71.25	78.75
9	100	95	81.67	88.33	92.5	95	88.33	91.67	72.5	72.5
10	90	97.5	88.33	96.67	97.5	100	90	88.33	78.75	81.25
11	97.5	100	91.67	96.67	90	95	83.33	83.33	67.5	68.75
12	92.5	92.5	86.67	90	97.5	97.5	85	96.67	72.5	77.5
13	95	100	73.33	86.67	92.5	97.5	85	93.33	72.5	73.75
14	90	95	83.33	93.33	90	100	88.33	93.33	72.5	76.25
15	97.5	100	91.67	100	97.5	92.5	91.67	85	71.25	73.75
Average	92	95.17	83.89	90.33	93	96.17	87.89	90.89	72.91	77.17
Time (s)	0.2	0.04	1.58	0.21	0.33	0.06	2.84	0.26	4.61	0.45

**Table 5:** Mean (standard deviation) value of topological features for all subjects in the SEED.

Feature	Sad	Neutral	Happy
SACC	1.82 (0.12)	1.74 (0.14)	1.95 (0.14)
SACT	0.28 (0.06)	0.25 (0.05)	0.34 (0.07)
STTC	0.09 (0.03)	0.07 (0.02)	0.12 (0.03)
SAC	51,858.37 (3542.62)	49,995 (3674.12)	54,838.94 (4003.06)
SDCP	35.18 (2.06)	33.84 (2.04)	37.05 (2.09)

**Table 6:** Mean (standard deviation) value of topological features for all subjects in the HIED.

Feature	Sad	Neutral	Happy	Afraid
SACC	2.56 (0.26)	2.5 (0.26)	2.61 (0.25)	2.55 (0.27)
SACT	0.63 (0.10)	0.61 (0.10)	0.66 (0.09)	0.62 (0.09)
STTC	0.26 (0.05)	0.24 (0.05)	0.27 (0.05)	0.25 (0.05)
SAC	69,351.45 (4432.61)	68,223.98 (4624.55)	70,901.06 (4348.68)	69,233.29 (4042.20)
SDCP	44.598 (2.69)	43.96 (2.77)	45.48 (2.51)	44.88 (2.52)

**Table 7:** Comparison with existing studies based on the SEED.

References	Feature	Classifier	Accuracy
Zheng and Lu [19]	DE, PSD, DASM, RASM, DCAU	SVM	86.65%
Kouti et al. [37]	iCoh connection feature	SVM	83.84%
Xu et al. [38]	HOC, FD, band power, DE	SVM	81.90%
Yan et al. [39]	DE, PSD, DASM, RASM, DCAU	Spatio-temporal Graph Bert network	83.20%
Sun et al. [40]	RMS + DE	RF	88.93%
Ours	Topological Features	XGBoost	90.30%
Ours	Topological Features + DE	XGBoost	92.00%

## Data Availability

The data is unavailable due to privacy or ethical restrictions.
